# A Metaverse Program to Prevent E-Cigarette Use Among Adolescents: Pilot Mixed Methods Study

**DOI:** 10.2196/65817

**Published:** 2026-06-24

**Authors:** Hyunmi Son, Gyumin Han

**Affiliations:** 1 College of Nursing, Research Institute of Nursing Science, Pusan National University, 49, Busandaehak-ro, Mulgeum-eup, Yangsan-si, 50612, Republic of Korea, 82 51 510 8347

**Keywords:** e-cigarette, adolescent, metaverse, pilot study, smoking prevention, focus group, health education, user engagement

## Abstract

**Background:**

E-cigarette use among adolescents presents challenges distinct from traditional smoking issues. The perception that e-cigarettes are less harmful and their distinctive appeal as a means of sensation seeking among adolescents suggest that traditional smoking prevention programs may not guarantee effectiveness in preventing e-cigarette use. Therefore, there is an urgent need for educational interventions to prevent adolescents from using e-cigarettes.

**Objective:**

This pilot study aimed to assess the potential of the Meta Nodam program, a novel metaverse-based educational initiative to enhance adolescents’ resistance to e-cigarette use and increase their engagement in preventive education.

**Methods:**

The Meta Nodam program was developed on the Roblox platform, designed to promote autonomous engagement and health-related decision-making among adolescents. The study included 120 participants in a 2-session intervention over 2 weeks, each session lasting 45 minutes. Changes in key cognitive outcomes (e-cigarette use intentions, attitudes, norms, self-efficacy, and interest development) were examined using pre- and postintervention surveys. In addition, focus group interviews with 10 participants were conducted to gain deeper insights into participants’ experiences.

**Results:**

Quantitative analysis showed a significant within-group increase in e-cigarette–related self-efficacy (*t*_119_=–3.171; *P*=.002), whereas no statistically significant pretest-posttest differences were observed in intentions (*t*_119_=–0.846; *P*=.40) or attitudes (*t*_119_=−0.080; *P*=.94). Qualitative findings explained and expanded these results by indicating that immersive metaverse experiences made e-cigarette–related risks more tangible, encouraged peer discussion, and promoted reflection on e-cigarette–related decision-making. Participants also described the program as engaging and reported interest in further exploring information about the health risks of e-cigarette use.

**Conclusions:**

Owing to the short duration of the metaverse experience, limited to 2 sessions over 2 weeks, the program did not measurably alter adolescents’ attitudes or intentions regarding e-cigarette use. However, it was associated with enhanced e-cigarette–related self-efficacy and demonstrated the applicability, usefulness, and attractiveness of the metaverse program. This study highlights the potential of digital platforms such as the metaverse for health education, emphasizing user engagement, interaction, and experiential learning in preventive education. Future research should consider broader participant demographics and more robust experimental designs to more rigorously evaluate the potential of such interventions.

## Introduction

Recent global statistics indicate that e-cigarettes have the highest use rate among tobacco-related products used by adolescents [1]. E-cigarettes are linked to health risks such as myocardial infarction, upper respiratory irritation, immune system disruption, and reduced fertility [1,2]. The use of e-cigarettes by adolescents demonstrates a “gateway effect” that leads to other health issues [3]. Notably, the inclusion of nicotine not only induces addiction but also impairs brain development in adolescents [4], and the capability of users to adjust the quantity of nicotine solutions puts them at high risk of nicotine exposure. In South Korea, national data also show that adolescent e-cigarette use remains a pressing concern. Although the prevalence of conventional cigarette smoking among Korean adolescents continues to decline, e-cigarette use shows an opposite trend. According to the 2023 Korea Youth Risk Behavior Web-based Survey, the rate of e-cigarette use among adolescents steadily increased from 1.9% in 2020 to 2.9% in 2021, reaching 3.3% in 2022 [5]. These consistently rising rates underscore the necessity for targeted prevention efforts within this population.

Existing tobacco-related youth prevention programs are focused primarily on conventional tobacco products [6]. Although some studies have included e-cigarettes in broader adolescent behavioral interventions [7,8], there has been no focus on e-cigarettes. A systematic review of interventions for the prevention of youth tobacco and nicotine use revealed that no studies targeted e-cigarettes [9]. This underscores a critical oversight as most existing programs encompass general tobacco use rather than focusing on e-cigarette use. Although recent strategies targeting e-cigarette use have evolved, they often repurpose traditional smoking prevention techniques [10]. Unlike traditional cigarettes, e-cigarettes foster a positive social norm around their use, largely due to perceptions of reduced harm and the customizable nature of flavors and scents, appealing to youth’s desire for personalization and variety. Additionally, the sleek, technology-oriented design of e-cigarettes, coupled with their discreet use and portrayal on social media, enhances their attractiveness and social acceptability among peers. This unique appeal, combined with the communal aspect of vaping, underscores the need for specifically tailored interventions that address the nuances of e-cigarette use among youth, challenge positive norms, and highlight the health risks involved.

Given adolescents’ growing perception of e-cigarettes as appealing [11], prevention efforts must address the key cognitive determinants of decision-making—specifically, attitudes, perceived social norms, and refusal self-efficacy—within the social contexts where vaping occurs. These cognitive factors are not isolated; they are shaped by multilevel influences, including peer behavior, school and family norms, retail access, and media exposure. Refusal self-efficacy, a concept rooted in the social cognitive theory by Bandura [12,13], refers to the belief in one’s capability to successfully decline e-cigarettes despite peer pressure or temptation.

To reinforce this efficacy, repeated mastery experiences in diverse contexts are essential. Metaverse environments are particularly well suited for this purpose, offering immersive, realistic, yet safe simulations of high-risk situations that are difficult to replicate in real life [14-16]. Within these virtual spaces, adolescents can practice refusal skills and observe how peer reactions and environmental cues influence their attitudes and norms through collaborative gameplay and problem-based scenarios. Therefore, developing metaverse-based educational programs that incorporate these multilevel influences into their design to specifically target changes in adolescents’ attitudes, norms, and self-efficacy represents a promising strategy for e-cigarette use prevention.

An examination of existing adolescent educational programs using the metaverse reveals that traditional lecture-based content is frequently transferred directly into virtual realms. However, this approach has been criticized for not fully exploiting the potential of metaverse environments, which offer significant freedom and diverse experiential possibilities [14,17]. Such programs often limit opportunities for real-life experiential learning and fail to leverage the extensive capabilities of the metaverse, especially in contexts where practically any scenario can be simulated [18]. Additionally, research on the actual experiences of adolescents within these highly interactive and immersive metaverse environments remains scarce [19]. To address this gap, a pilot study was conducted to investigate the potential of the metaverse for raising awareness and delivering preventive health education to adolescents. The immersive and interactive nature of metaverse environments uniquely supports experiential learning and the strengthening of refusal self-efficacy, crucial components for effective health education. This pilot study aimed to evaluate the feasibility and user engagement of an interactive metaverse program and examine preliminary within-group changes in key cognitive outcomes (self-efficacy, attitudes, norms, and intentions) related to e-cigarette use among adolescents.

## Methods

### Research Design

This study used an explanatory sequential mixed methods pilot design in which the quantitative phase was conducted first followed by a qualitative phase to provide a more in-depth understanding of the quantitative findings. In the quantitative phase, a single-group pretest-posttest design was used with 120 participants to evaluate the feasibility and acceptability of the intervention and assess preliminary changes in key cognitive outcomes. After the intervention and postintervention survey, qualitative data were obtained from 10 participants through focus group interviews to explore participants’ experiences within the metaverse and provide contextual insights into how the program influenced their perceptions of and attitudes toward e-cigarettes. The qualitative themes were then used to explain and expand upon the quantitative results. Integration occurred during the interpretation and presentation of the findings by linking quantitative outcomes with related qualitative themes.

### Development of Meta Nodam

The Meta Nodam program was developed to leverage the immersive capabilities of the metaverse, allowing adolescents to safely experience and navigate the multilevel factors influencing e-cigarette use within a simulated environment. The program was developed and implemented on the Roblox platform. The name Meta Nodam combines “Meta,” referencing its metaverse-based platform, with “Nodam,” a Korean term meaning “no tobacco,” reflecting the program’s purpose of promoting healthy decision-making regarding e-cigarette use.

The development of Meta Nodam was grounded in the multilevel determinants of adolescent e-cigarette use identified by Han and Son [20]. Recognizing that e-cigarette use is driven by a complex interplay of individual, interpersonal, and environmental factors, the program used the metaverse to simulate these multilevel influences within a safe, virtual space. While the intervention environment mirrored these broader contextual factors, the educational strategies were specifically designed to enhance individual-level cognitive determinants—attitudes, norms, and self-efficacy—as the proximal mechanisms for driving behavior change.

**Figure 1.**
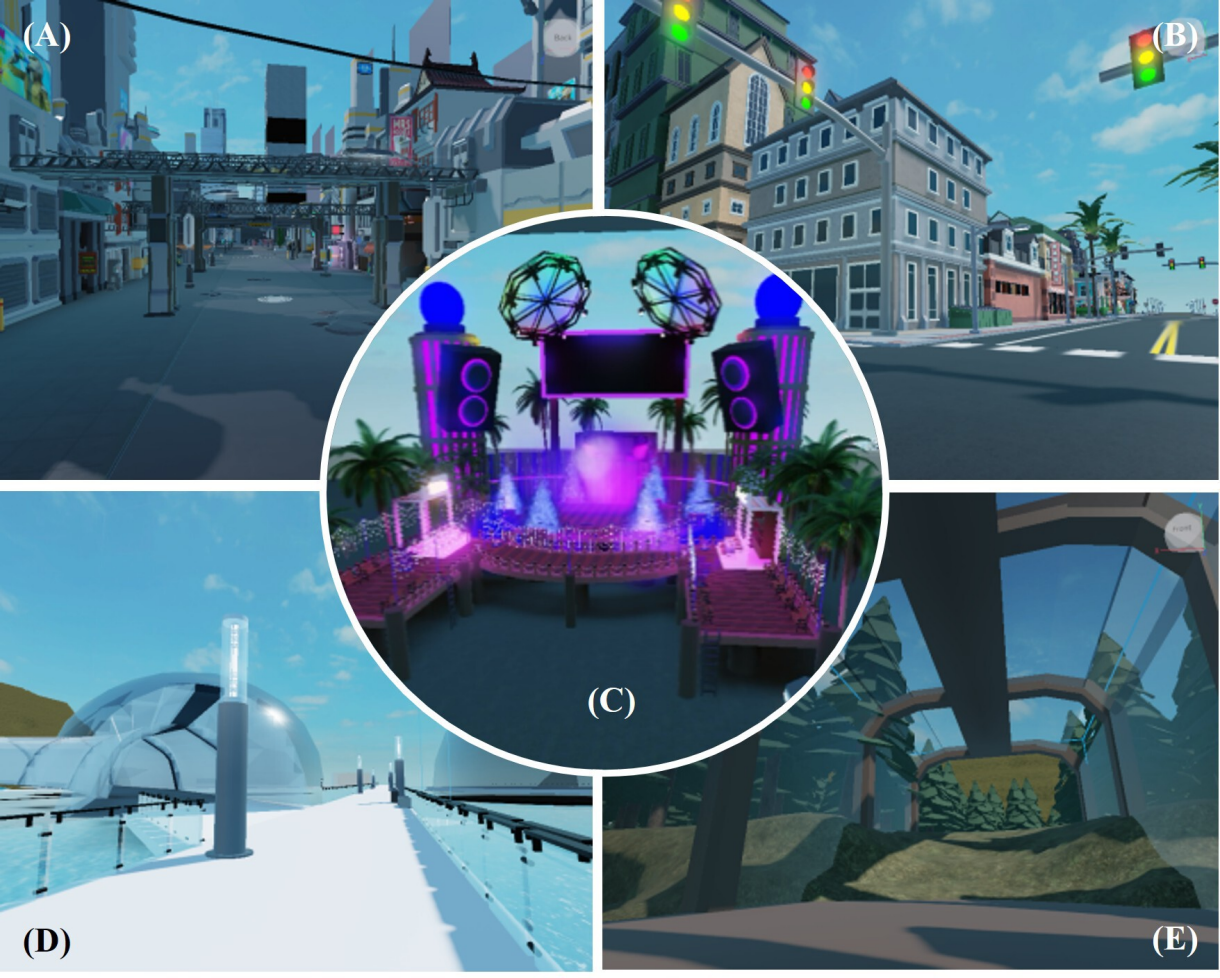
Screenshots of the Meta Nodam metaverse environment. The labeled panels show the interconnected virtual spaces used in the program: (A) Play Zone, (B) Living Zone, (C) Fashion Square, (D) Treatment Zone, and (E) Forest Zone.

The Meta Nodam program incorporated its learning objectives into a set of interconnected metaverse spaces that adolescents could explore. These included Fashion Square (Figure 1C), Play Zone (Figure 1A), Living Zone (Figure 1B), Forest Zone (Figure 1E), and Treatment Zone (Figure 1D). Each zone provided a distinct contextual experience relevant to how adolescents encounter e-cigarette–related cues in their daily lives. Fashion Square served as the central gathering space where participants interacted with peers’ avatars and expressed identity through customization, offering natural opportunities to observe social dynamics. The Play Zone and Living Zone reflected familiar social environments in which adolescents often encounter peer influence and normative cues. These spaces allowed adolescents to observe others’ behaviors and reflect on how social expectations shape their own decisions.

The Forest Zone incorporated a symbolic representation of the negative consequences of sustained e-cigarette use through encounters with zombies. This design provided an experiential but age-appropriate way to illustrate potential harms associated with nicotine use. Virtual e-cigarette vending machines were strategically placed throughout the Play, Living, and Forest Zones to create realistic decision-making points. When participants approached a machine, they were required to make a choice—interact with it or not. If they chose to interact, they were transported to the Treatment Zone, where their activities were temporarily restricted and they were informed about the consequences of the action. This mechanism encouraged participants to reflect on their decision and reinforced the link between choices and outcomes.

Nonplayer characters (NPCs) were integrated throughout the environment to deliver informational messages about health risks, model social reactions, and simulate peer influence. Some NPCs provided direct health-related information intended to support shifts in attitudes toward e-cigarettes. Others expressed approving or disapproving views of vaping, enabling participants to consider how social expectations and peer cues can shape normative beliefs. NPC conversations and scripted scenarios were carefully designed to resemble situations that adolescents may encounter in their everyday lives.

The program’s interactive components were strategically designed to function as pedagogical tools, transforming abstract health concepts into concrete virtual experiences. The combination of informational messages, symbolic representations, peer interactions, situational cues, and decision-making moments created an integrated experiential learning environment that supported the targeted cognitive constructs. Opportunities to make choices in ambiguous or socially pressured situations were included to strengthen self-efficacy by allowing adolescents to practice navigating tempting or uncomfortable scenarios within a safe virtual space. The structured yet exploratory nature of the metaverse world enabled repeated engagement with these learning opportunities while maintaining a sense of autonomy.

The program was delivered over 2 structured 45-minute sessions conducted in person in school classrooms. Students used laptops with stable internet connections to access the Meta Nodam environment simultaneously, allowing for real-time interactions with peers’ avatars. The first session focused on orientation, initial exposure, and exploration of the metaverse environment. It included a 20-minute prebriefing via Zoom (Zoom Video Communications) explaining the program’s objectives and instructions, followed by 20 minutes of exploration within the metaverse and a short debriefing. The second session was designed to provide further guided and unguided exploration, peer interaction, reflection on e-cigarette–related decision-making, participation in a fashion contest, and a concluding debriefing along with the postsurvey. Teachers monitored students’ activities through their own computers, providing technical support and ensuring safety while minimizing disruptions to the immersive experience.

### Pilot Test of Meta Nodam

The pilot test of the Meta Nodam program was conducted as a preliminary investigation among 120 girls’ middle school students. This sample size was determined using the G*Power program (version 3.1.9.7) based on a 2-tailed paired *t* test with an 80% power level and a significance level of .05. The effect size, set at 0.257, was based on the impact of a virtual reality game program aimed at preventing adolescent e-cigarette use [21]. To account for a potential dropout rate of approximately 20%, as observed in similar school-based smoking prevention programs [22], 120 participants were initially recruited.

The pilot included pre- and postintervention surveys and focus group interviews with 10 highly engaged students to gain qualitative insights into their experiences. These interviews were conducted in 2 groups, each consisting of 5 highly engaged students. Highly engaged students were selected because the qualitative phase of this pilot study aimed to obtain detailed feedback on program experience, usability, engagement, and areas for improvement. Students who had actively explored the metaverse environment were expected to provide more specific descriptions of program functions, immersive elements, difficulties, and suggestions for refinement. The 2-session, 90-minute format was selected to align with the regular class period structure of the participating middle school and examine the feasibility of implementing the program in a real school setting. As this was a pilot study, the program was intended to assess feasibility, acceptability, engagement, and preliminary cognitive responses rather than long-term cognitive or behavior change. Surveys were administered before the first session and after the second session, each taking approximately 6 to 9 minutes to complete.

### Measurement and Data Collection

The first section of the questionnaire assessed self-efficacy in resisting e-cigarette use, a key factor in understanding participants’ confidence in handling temptations related to e-cigarette use. This was measured using a tool adapted from Condiotte and Lichtenstein [23]. This scale demonstrated good internal consistency, with Cronbach α values of 0.970 (pretest) and 0.973 (posttest). The next section focused on e-cigarette–related characteristics, particularly the influence of peers’ e-cigarette use, which is a critical determinant in adolescent behavior. The aim of this section was to capture the social environment of the participants regarding e-cigarette use. To further categorize the participants, e-cigarette use patterns were assessed by inquiring about both lifetime and recent e-cigarette use, thereby classifying participants as lifetime or current e-cigarette users.

Participants’ intentions toward future e-cigarette use were evaluated using modified questions from Pierce et al [24-26] to gauge their susceptibility to e-cigarette use. The Cronbach α for this scale was 0.748 (pretest) and 0.840 (posttest).

Attitudes toward e-cigarettes were measured using an instrument adapted from de Vries et al [27] and modified by Kim and Choi [28]. This section of the questionnaire explored the participants’ perceptions of the benefits and risks associated with e-cigarette use. The Cronbach α for this scale was 0.921 (pretest) and 0.842 (posttest). Additionally, e-cigarette–related norms were assessed by adapting a tool from Primack et al [29] to evaluate the perceived social acceptance and prevalence of e-cigarette use within the participants’ social circles. The Cronbach α for this measure was 0.617 (pretest) and 0.665 (posttest).

After the intervention, the survey included additional items to evaluate the impact on participants’ interest development using an instrument adapted from Wang and Adesope [30]. The subdomains of this instrument assessed various facets of interest development, including triggered situational interest, maintained situational interest, emerging individual interest, and well-developed individual interest, each rated on a scale from 1 to 7, where 1 indicated the lowest level of interest and 7 indicated the highest. The Cronbach α for this scale was 0.941 (posttest).

To complement the quantitative data, qualitative insights were gathered through focus group interviews with participants who showed high engagement in the program and were willing to discuss their experiences extensively. This approach was used to obtain rich, experience-based feedback on program usability, immersion, perceived educational value, and areas for refinement. These interviews began with the following broad question: “How was your experience with the Meta Nodam program?” This open-ended approach allowed participants to share their detailed experiences freely, providing a deeper understanding of the program’s impact from their perspective. The researchers facilitated natural discussions among participants, asking clarifying questions only when necessary to confirm or explore specific points in greater depth.

### Ethical Considerations

This study was approved by the institutional review board of Pusan National University (PNU IRB/2022_165_HR). Participants who voluntarily expressed interest were thoroughly informed about the study’s purpose, content, procedures for withdrawal, privacy protections, and compensation measures in case of research-related harm. As all participants were under 18 years of age, written informed consent was obtained from each adolescent participant as well as their legal guardians through a detailed explanatory letter and consent form. Participants whose guardians declined consent or who chose not to take part were allowed to opt out without any consequences; however, apart from students who were absent from school, there were no cases of withheld consent in this study. Participants received a snack voucher worth KRW 1500, approximately US $1.10 (KRW 1=US $0.00073 as of June 11, 2026), after completing each of the pre- and postintervention surveys as a token of appreciation for their time. This compensation was not linked to their survey responses. The consent procedure ensured informed and voluntary participation, adhering to the highest standards of research ethics and safeguarding the rights and well-being of adolescent participants and their guardians.

### Data Analysis

For quantitative data, statistical analysis was performed using SPSS (version 23; IBM Corp). Descriptive statistics were used to assess demographic details, and paired *t* tests were used to compare pre- and postintervention measures of e-cigarette–related cognitive outcomes, including self-efficacy, intentions, attitudes, and normative beliefs, with the significance threshold set at a *P* value of less than .05.

Content analysis was carried out on qualitative data from the focus group interviews to extract themes and patterns. All discussions were audio recorded, transcribed verbatim, and subjected to an inductive content analysis process. Two researchers independently performed line-by-line coding, compared their codebooks, and resolved discrepancies through discussion until full agreement was reached. Codes were then grouped into subcategories and distilled into overarching themes that reflected common perceptions of program usefulness, realism, and perceived changes in refusal confidence. Data saturation was reached after conducting 2 focus group interviews (totaling 10 participants) as no new themes emerged. An audit trail documenting analytic decisions and periodic peer debriefings enhanced dependability, whereas member checking with 30% (3/10) of the participants confirmed that the derived themes accurately represented their views.

The quantitative and qualitative findings were then integrated at the interpretation stage. Specifically, quantitative results were first reviewed for each outcome domain, and related qualitative themes were subsequently linked to these outcomes to determine whether the qualitative findings explained, complemented, or expanded the quantitative findings. This integrated interpretation was used to organize the Results section around preliminary cognitive responses and program experiences.

## Results

### Participant Characteristics

This study involved female middle school students, none of whom had ever used or were currently using e-cigarettes. Regarding friends’ e-cigarette use, 75% (90/120) reported no use among their peers, whereas 25% (30/120) acknowledged that some friends used e-cigarettes. Among the 10 students who participated in the focus group interviews, none had personal experience using e-cigarettes, although 3 (30%) mentioned having friends who used e-cigarettes.

### Engagement and Participation Experience in the Metaverse Program

The overall postintervention interest development score averaged 5.59 (SD 0.97), suggesting that participants were engaged with the program content. Among interest subdomains, triggered situational interest was highest (mean 6.11, SD 1.07), followed by maintained situational interest (mean 5.86, SD 1.23), well-developed individual interest (mean 5.55, SD 0.85), and emerging individual interest (mean 5.33, SD 1.10; Table 1). These findings should be interpreted as exploratory, reflecting short-term responses observed within the context of a brief, 2-session pilot program.

**Table 1.** Pretest-posttest changes in e-cigarette–related cognitive outcomes and interest development from the Meta Nodam program.

Variable and subcategory	Pretest, mean (SD)	Posttest, mean (SD)	*t* test (*df*)	*P* value
Self-efficacy (0-10)	9.65 (0.91)	9.86 (0.58)	−3.171 (119)	.002
Intention (4-16)	15.57 (1.06)	15.64 (1.07)	−0.846 (119)	.40
Attitudes (12-60)	54.68 (7.43)	54.74 (6.55)	−0.080 (119)	.94
Normative belief				
Perceived prevalence (0-300)	125.92 (50.03)	120.25 (52.64)	1.695 (119)	.09
Approval by parents or peers (3-12)	4.52 (2.34)	4.78 (2.97)	−0.922 (119)	.36
Popularity among the successful or elite (4-16)	12.78 (1.95)	12.77 (2.45)	0.086 (119)	.93
Interest development (1-7)		5.59 (0.97)		
Triggered situational interest	—^a^	6.11 (1.07)	—	—
Maintained situational interest	—	5.86 (1.23)	—	—
Emerging individual interest	—	5.33 (1.10)	—	—
Well-developed individual interest	—	5.55 (0.85)	—	—

a Not applicable.

The focus group interviews also showed that the metaverse environment enhanced engagement and learning compared with traditional methods. Participants described the program as enjoyable, gamelike, and immersive, which helped maintain their interest in e-cigarette use prevention education.

Participants noted that, unlike traditional smoking prevention education, which they found boring, the metaverse program was enjoyable and encouraged active participation. The program’s playful approach was key to maintaining interest:

We usually play in the metaverse for fun or as a hobby. But this time, we first encountered it through an educational program. At first, I thought it would be boring like the typical education we received in elementary school, where they just keep telling us facts and why smoking is bad. But this program turned education into a game, which made it much more interesting and engaging for us.

[Participant 2]

Participants also reported sustained engagement and broadened interest beyond the assigned sessions. The program was so engaging that some participants continued to interact with it even after the sessions ended, sparking broader educational interests beyond e-cigarettes:

After just one session, I found the storyline to be really extensive and interesting. So, I kept playing two or three more times over the week. The game had a lot of addicts, and shooting them with a gun to deal with them was surprisingly fun. It made me curious about why they became addicted in the first place, which encouraged me to look into it more.

[Participant 3]

### E-Cigarette Refusal Self-Efficacy

Analysis of the quantitative data indicated a statistically significant within-group increase in self-efficacy related to making decisions about e-cigarette use, with scores rising from 9.65 (SD 0.91) before participation to 9.86 (SD 0.58) after participation (*t*=–3.171; *P*=.002; Table 1).

The qualitative findings provided contextual insights into this increase by showing that participants did not experience e-cigarette–related situations as abstract health messages but as personally relevant scenarios through their avatars. In particular, the avatar-based experience appeared to help participants place themselves in virtual situations involving e-cigarette use and consider how they might respond to similar temptations or peer-related situations in real life:

Experiencing the effects of smoking in the metaverse felt more real than just hearing about it. The metaverse experience was more impactful because my avatar in the game represented me. It felt like I was the one smoking and being taken to a confined space, which made the experience very novel and engaging.

[Participant 6]

The postintervention survey also appeared to provide an additional opportunity for participants to reflect on how they would respond to e-cigarette offers from peers. Rather than only recalling information about e-cigarette risks, participants described reconsidering their own behavioral responses in peer-related situations, including refusing use and discouraging peers from using e-cigarettes:

When I was filling out the survey, there were many question items like “I won’t smoke even if a friend offers.” Seeing these question items made me think about my actions. Now, if a friend smokes, I’ll stop them and make sure I don’t join in.

[Participant 1]

### Attitudes and Intentions Toward E-Cigarettes

No statistically significant pretest-posttest differences were observed in intentions, attitudes, or normative beliefs (Table 1). However, the qualitative findings suggested that participants developed greater awareness of e-cigarette–related risks and reflected on how e-cigarette use could affect them and their peers.

Participants described the metaverse scenarios as making the risks and consequences of e-cigarette use more vivid. In particular, the symbolic portrayal of addiction appeared to help participants recognize the difficulty of escaping e-cigarette dependence:

I remember the zombies saying things like “help me,” which made me realize how hard it must be to escape e-cigarette addiction. It really showed how difficult it can be to break free.

[Participant 8]

Participants also reported that the program created opportunities for more open conversations with peers about the risks of e-cigarettes. These discussions appeared to extend participants’ risk awareness beyond individual reflection to peer-related communication and prevention-oriented dialogue, although such changes were not captured as statistically significant pretest-posttest changes in attitudes, intentions, or normative beliefs:

First of all, I think the biggest change was in our awareness. In the metaverse, when we finish an activity, we talk to our friends about it. For example, we might say, “Did you know about the dangers of e-cigarette usage?” This creates an atmosphere where we can comfortably talk about these issues. If someone jokes about a friend who might be an e-cigarette user, we can now openly say, “Don’t use it; it’s not good for you.” Before, everyone would whisper and talk secretly, but now, since we all learned together and understand how bad it is, it’s more effective when several friends talk about it together. This has been the biggest real-world change.

[Participant 2]

### Metaverse Program Usability and Refinement

Focus group interviews provided feedback on the usability of the metaverse program and areas for refinement. Participants identified the need for clearer guidance, more realistic and direct educational content, increased interaction, and longer session duration.

Participants suggested the addition of a guide or tutorial for the metaverse environment, including a mini map, to enhance their experience and ease of use:

At first, I didn’t know what was going on at all. It would be helpful to have some kind of tutorial or help guide. Also, having a mini-map on the screen would be great to help navigate the structure, maybe placed in the corner.

[Participant 4]

Participants recommended making the virtual environment more realistic and incorporating more direct educational messages about e-cigarettes to increase the impact of the program:

I think it would be better if there was more content about e-cigarettes. Like, when you get stuck in the treatment room and have to talk to the NPC, it would be cool if you got more info about e-cigarettes at that point. Maybe even add a quiz or something.

[Participant 1]

When I go to the convenience store near school with my friends, there are so many e-cigarette ads that really stand out. It’s like They are made for students like us to see. If you could show scenes like this in the metaverse, I think it would help us realize the dangers of e-cigarettes.

[Participant 3]

Participants also expressed a desire for increased interaction with NPCs and students from other schools, as well as a longer program duration to fully explore the metaverse:

It would be great if we could interact with the NPCs. Besides that, I think it would be beneficial if this kind of game-based smoking prevention program could be implemented in other schools as well, not just ours.

[Participant 5]

The teacher explained everything well, but I felt like two sessions were not enough. It would have been better if the program had been longer.

[Participant 2]

## Discussion

### Principal Findings

This pilot study of the Meta Nodam program provided preliminary insights into how a metaverse-based intervention may support adolescents’ engagement, awareness, and cognitive readiness to resist e-cigarette use. The most notable quantitative finding was a statistically significant within-group increase in e-cigarette refusal self-efficacy. This finding was contextualized by the qualitative results, which showed that participants experienced e-cigarette–related situations as personally relevant through their avatars and reflected on how they might respond to peer offers or temptations in real life.

This improvement in self-efficacy may be related to the pedagogical strategies embedded within the metaverse environment. According to self-efficacy theory [12,13], mastery experiences are an important source of confidence. Meta Nodam was designed to provide simulated decision-making experiences in realistic but safe scenarios, allowing students to encounter e-cigarette–related cues and consequences without real-world risks. Qualitative findings suggested that the avatar-based experience made these situations feel personally meaningful and prompted participants to consider their own responses to similar situations. These findings suggest a possible pathway through which virtual rehearsal and reflection may support e-cigarette refusal self-efficacy.

The observed pattern aligns with earlier studies showing that virtual and immersive environments can effectively enhance adolescents’ knowledge and confidence [21]. Unlike traditional didactic education, which often relies on passive information transfer, the metaverse allowed for situated learning, where students actively navigated peer influence and environmental cues. Accumulating evidence supports this approach, suggesting that immersive or virtual reality–based lessons elicit higher engagement and learning outcomes than traditional methods among secondary school learners [14,16]. In this respect, Meta Nodam offers a promising strategy to supplement conventional prevention approaches by providing digital native youth with a safe, interactive space to build behavioral competence.

The absence of statistically significant changes in attitudes and intentions regarding e-cigarette use contrasts with findings from some earlier prevention programs [7,28]. However, several contextual factors may help explain this pattern. Many participants in this study already reported low intention to use e-cigarettes and generally negative attitudes toward vaping before the intervention, suggesting limited room for measurable shifts. Moreover, the program consisted of only 2 short sessions, which may not have been sufficient to influence more stable cognitive constructs. Nonetheless, qualitative data revealed that many participants reflected more deeply on vaping risks, expressed heightened awareness, and reported increased comfort discussing e-cigarette issues with peers following the program. These qualitative impressions may reflect subtle early shifts that were not detectable in the quantitative measures over such a brief time frame. As a pilot study, these findings highlight the need for further research with longer or more intensive implementations and more diverse samples to more fully examine the potential for cognitive and attitudinal shifts.

The evaluation of interest development scores indicated strong engagement and intrinsic motivation among participants, consistent with outcomes observed in prior metaverse-based educational programs [31]. Triggered situational interest showed the highest postintervention levels, suggesting that the novelty and interactive nature of the metaverse environment successfully captured students’ attention. This finding aligns with those of research demonstrating the motivational benefits of gamified or interactive learning environments [18,32]. Participants’ qualitative responses also supported this as several reported exploring the program beyond assigned sessions and described becoming curious about issues related to e-cigarette use. The program’s autonomy-enhancing features, symbolic representations, and opportunities for experiential learning may have contributed to these patterns. Additionally, the implementation of scenario-based role-play allowed students to actively rehearse refusal skills within contexts that closely mirrored real-life situations [21,32]. This practical application likely contributed to the students’ perceived increase in confidence in managing vaping temptations.

Regarding the program design, participants differentiated Meta Nodam from existing metaverse educational platforms that often replicate traditional classroom environments, such as those on Gather Town [33]. In contrast, this program was appreciated for its experiential and narrative-driven elements that transcended simple knowledge transmission. However, feedback indicating a desire for more explicit educational content suggests that future iterations should aim for a refined balance between open-ended exploration and structured instructional density. This aligns with user-centered design principles [34], which emphasize that harmonizing learner autonomy with clear educational goals is essential for maximizing both engagement and learning efficacy.

Successful implementation also relied heavily on the educator’s role. Teacher-led prebriefing sessions were identified as a critical component for orienting students and framing the experience as an educational intervention rather than purely recreational. As real-time interventions during the simulation can disrupt immersion [14], providing clear guidance beforehand proved to be the most effective strategy. However, adopting such high-technology tools imposes practical demands on educators, including the need for technical proficiency and additional preparation time [14,35], highlighting the necessity for adequate infrastructure and teacher support systems.

This pilot study offers preliminary indications that metaverse-based approaches may be promising for supporting adolescents’ awareness and confidence regarding e-cigarette use. The immersive and interactive nature of the program may help create opportunities for reflective learning and peer dialogue. However, several limitations must be considered. First, as this study was designed as a pilot trial to evaluate feasibility and immediate preliminary responses, the findings reflect short-term changes only. Therefore, these results should be interpreted as exploratory evidence rather than definitive proof of the program’s long-term effectiveness. The brief 2-session, 90-minute intervention exposure was an important limitation and may have been insufficient to produce measurable changes in relatively stable cognitive constructs such as attitudes and intentions. The single-gender, single-school sample also restricts generalizability to more diverse populations. Technical requirements, teacher workload, and system stability pose additional considerations for broader implementation. Furthermore, initial program deployment revealed operational challenges such as software bugs and the breadth of the virtual world relative to available time, suggesting opportunities to streamline content in future iterations. Addressing these limitations—through longer interventions, additional or booster sessions, repeated role-play or refusal practice, stronger integration of structured educational content, improved technical support, follow-up assessments, and multisite evaluation—will be essential to fully understand the potential role of metaverse-based programs in adolescent tobacco use prevention.

### Conclusions

This pilot study suggests that the Meta Nodam program may offer an engaging and interactive means of supporting adolescents’ awareness and confidence related to e-cigarette use. By situating educational content within a metaverse environment, the program provided opportunities for students to explore realistic social situations, interact with peers, and reflect on vaping-related decisions in a safe and immersive setting. While no measurable changes were observed in attitudes or intentions during this short 2-session format, participants demonstrated increased self-efficacy and described positive engagement with the program’s activities.

Given its exploratory nature, the findings should be interpreted cautiously. Nonetheless, the results indicate that metaverse-based environments may hold promise as a complementary tool within school-based prevention efforts, particularly for digital native adolescents who may benefit from interactive and experience-driven learning. Further refinement and evaluation—including longer program duration, expanded educational content, and more diverse samples—are needed to more fully understand how metaverse-based programs can support adolescents’ decision-making and contribute to broader strategies aimed at preventing e-cigarette use among youth.

## Abbreviations

NPC: nonplayer character
